# Improving IUI success by performing modified slow-release insemination and a patient-centred approach in an insemination programme with partner semen: a prospective cohort study

**DOI:** 10.52054/FVVO.13.4.045

**Published:** 2021-12-30

**Authors:** W Ombelet, I Van der Auwera, H Bijnens, J Onofre, C Kremer, L Bruckers, G Mestdagh, R Campo, N Dhont

**Affiliations:** Genk Institute for Fertility Technology, Department of Obstetrics and Gynaecology, Schiepse Bos 6, 3600 Genk, Belgium; Faculty of Medicine and Life Sciences, Hasselt University, Martelarenlaan 42, Hasselt, Belgium; Interuniversity Institute for Biostatistics and Statistical Bioinformatics, Data Science Institute, Hasselt University, Martelarenlaan 42, Hasselt, Belgium.

**Keywords:** clinical pregnancy rate, infertility, intrauterine insemination, IUI, homologous, patient-centred care, slow-release insemination

## Abstract

**Background:**

Pregnancy rates after in vitro fertilisation (IVF) treatment continue to improve, while intrauterine insemination (IUI) programmes show no such trend. There is a need to improve success rates with IUI to retain it as a viable option for couples who prefer avoiding IVF as a first line treatment.

**Objective:**

To investigate if a modified slow-release insemination (SRI) increases the clinical pregnancy rate (CPR) after intrauterine insemination (IUI) with partner semen.

**Materials and Methods:**

This was a prospective cohort study in a Belgian tertiary fertility centre. Between July 2011 and December 2018, we studied data from an ongoing prospective cohort study including 989 women undergoing 2565 IUI procedures for unexplained or mild/moderate male infertility. These data were analysed in order to study the importance of different covariates influencing IUI success. Generalised estimating equations (GEEs) were used for statistical analysis. Results of two periods (2011-2015, period 1 and 2016-2018, period 2) were examined and compared. From January 2016 (period 2) onwards, a standardised SRI procedure instead of bolus injection of sperm was applied. The primary outcome parameter was the difference in clinical pregnancy rate (CPR) per cycle between period 1 (bolus IUI) and period 2 (modified SRI). Secondary outcome results included all other parameters significantly influencing CPR after IUI.

**Results:**

Following the application of modified SRI the CPR increased significantly, from 9.03% (period 1) to 13.52% (period 2) (p = 0.0016). Other covariates significantly influencing CPR were partner’s age, smoking/ non-smoking partner, BMI patient, ovarian stimulation protocol and Inseminating Motile Count (after semen processing).

**Conclusion:**

The intentional application of modified slow-release of processed semen appears to significantly increase CPRs after IUI with homologous semen. Future studies should investigate whether SRI, patient-centred measures, or a combination of both, are responsible for this improvement.

## Introduction

The rationale behind intrauterine insemination (IUI), with or without ovarian stimulation (OS), is to increase the gamete density at the site of fertilisation.

IUI is less stressful, less invasive and less expensive than in vitro fertilisation (IVF) and evidence from different patient populations supports IUI as a first- line treatment option over IVF in selected cases of unexplained and mild/moderate male infertility (Bensdorp et al., 2015; Tjon-Kon-Fat et al., 2015; Bahadur et al., 2016; Ombelet et al., 2017; Farquhar et al., 2018).

On the other hand, because of rising success rates upon IVF/ICSI treatment, translating into better implantation rates per embryo, one might question the future role of IUI.

Increasing implantation rates in IVF/ICSI programmes are the result of multiple improvements including on laboratory air quality (Scorio et al., 2021), quality control (Agarwal et al., 2017) and the overcoming of technical challenges of embryo(s) selection and transfer by use of state-of-the-art close incubation systems, ultrasound guidance and soft catheters (Mains and Van Voorhis, 2010).

Above this, the main complication associated with IVF, namely high order multiple births, has declined significantly as emphasis on the value of transferring less embryos have resulted in an increased use of single embryo transfer (SET).

In a recently published ‘fertile battle’ article (Ombelet et al., 2020), arguments favouring IVF over IUI as a first-line treatment in unexplained infertility were put forward, arguing that changes in IVF practices and regimens have caused a significant reduction in complications and improvement in patient acceptability. Pregnancy rates after IVF continue to improve, while IUI programmes show no such trend. If we are unable to increase the IUI success rate, IVF will become the favoured first-line treatment for most causes of infertility in heterosexual couples (Moolenaar et al., 2015).

However, a first-line IVF strategy does not always take into account the couple’s or women’s treatment preferences. When given a choice, a substantial number of patients will prefer IUI because it is perceived as a more natural and less aggressive procedure.

Nevertheless, if IUI is to remain a useful first- line treatment option in unexplained and mild male infertility, the delivery rate per cycle needs to improve without increasing the multiple pregnancy rate and risk of complications such as ovarian hyperstimulation syndrome (OHSS).

Previous reports showed that a slow-release intrauterine insemination (SRI) might improve the pregnancy rate when compared to bolus IUI. In these studies, a commercially available product was used (Grasby auto-syringe driver or the EVIESlow- Release Insemination Pump) (Muharib et al., 1992; Marschlek et al., 2017, 2020).

In this study, we aimed to investigate the difference in clinical pregnancy rates in two time periods: before 2016 (period 1) bolus inseminations were per performed by medical doctors, from 2016 onward (period 2) midwifes performed modified slow-release inseminations. This new strategy of performing IUI was the only intervention parameter that differed between both periods.

## Material and methods

These data are part of a prospective observational cohort study performed at the Genk Institute for Fertility Technology (Thijssen et al., 2017). We studied the medical records of 989 sub-fertile couples with a total of 2565 inseminations with partner’s semen. Institutional Review Board approval was obtained (number 13/054U). All inseminations were performed between 1 July 2011 and 31 December 2018. By means of a questionnaire, during the 20 minutes of mandatory bed rest a midwife noted details on possible contributing factors affecting IUI success rates. In period 1 all inseminations were done within 5 to 10 seconds (bolus injection). From 2016 onward a modified slow-release insemination (SRI, between 45 and 60 seconds) was performed.

### Patient selection

All couples had been trying to conceive unsuccessfully for at least one year. Prior to IUI treatment, female patients were subjected to an infertility work-up, including medical history, physical examination, pelvic ultrasound, and serum hormone assays. A hysterosalpingography (HSG) or saline contrast hysterosalpingo-foam sonography (HyFoSy) was used to assess the uterine cavity and tubal patency. Where tubal or uterine abnormality was suspected, a hysteroscopy and/or laparoscopy was performed. In all men, one or two sperm examinations were performed according to the World Health Organization (WHO) guidelines (Cooper et al., 2010; WHO, 2010).

Couples suffering from unexplained infertility and mild or moderate male factor infertility with at least one patent fallopian tube were considered eligible for IUI treatment. The definition of mild/moderate infertility in this study applied for single, double or even triple semen abnormalities (oligozoospermia: < 14 mill/ml, asthenozoospermia: progressive motility: < 32 %, teratozoospermia: morphology: < 5%). IUI was only performed in couples with a partner’s IMC (inseminating motile count or number of motile sperm after sperm processing) above 1 million (Thijssen et al., 2017).

### Covariates

Beside the duration of insemination (bolus versus SRI) the following parameters were investigated: female and male age (years), smoking (non- smoking, 1–14 cigarettes a day, ≥15 cigarettes a day), BMI (kg/m 2 ), primary/secondary infertility, ovarian stimulation method, day 0 (day of ovulation triggering) oestradiol (ng/l) and progesterone (µg/l) levels, human chorionic gonadotrophin (hCG)- insemination time interval (hours), and sperm quality parameters [i.e. volume (ml), concentration (million/ml), total count (million), motility grade A (%), progressive motility (%), morphology (%), and post-processing inseminating motile count or IMC (million).

### Ovarian stimulation

As recommended by Cohlen et al. (2018) we used ovarian stimulation with clomiphene citrate (CC) or human menopausal gonadotrophins (hMG)/ recombinant FSH (rFSH) protocols in all our cases of unexplained infertility or oligo-/anovulation. Natural cycle IUI was performed in a minority of cycles (17%), mostly on demand of the patients provided menstrual cycles were regular. In case of ovulatory problems, we are obliged to use CC as a first-line treatment according to the Belgian law. When no pregnancy occurred after 3 trials of CC stimulation, whether or not with IUI, hMG or recFSH is reimbursed by the government. With the CC protocol, a single dose of clomiphene citrate (50 mg or 100 mg; Clomid ® , Sanofi, Belgium) was administered from days 3 until day 7. hMG and/ or rFSH (Menopur ® , Ferring, Belgium; Puregon ® , MSD, Belgium) was administered in a minimal dose step-up regimen, starting off with 50 IU or 75 IU on day 3 of the cycle. Follicular ultrasonography and serum oestradiol determinations were carried out on day 8–9 of the cycle and thereafter every other day. HCG 5000 IU injection (Pregnyl ® , MSD, Belgium) was given to trigger ovulation when the average diameter of the dominant follicle was 18 mm or more. If three or more follicles of at least 15 mm were present, the cycle was cancelled, and protected intercourse was advised.

### Semen examination and preparation

On the day of insemination, the semen sample was obtained through masturbation and collected in a sterile cup after a 2–4 day abstinence period. Within 1 h of production and after liquefaction at room temperature, the specimen was examined for initial volume, concentration, and progressive motility according to World Health Organization (WHO) guidelines (Cooper et al., 2010; WHO, 2010). The semen sample was washed to free from seminal fluid through double-density gradient (40% and 80%) with PureSperm® (PureSperm® 40/80, Nidacon), according to the manufacturer’s instructions (Nidacon International AB, Mölndal, Sweden). The IMC was determined after spermatozoa preparation by multiplying the percentage of hyperactive and grade A motility spermatozoa by sperm volume (1 ml) and concentration. Morphology scores were adapted from the first semen examination in the diagnostic phase.

### Intrauterine insemination: From bolus IUI to modified slow-release IUI

IUI was performed at 20–30 hours post-hCG. Until December 2015 the inseminations were performed by medical doctors (trainees or gynaecologists). Making use of a 1 ml syringe (VWR, Leuven, Belgium), the catheter (Gynétics Medical Products, Lommel, Belgium) was filled with 0.3 ml of prepared semen sample. The processed semen sample was gently injected over about 5 – 10 seconds (bolus injection). From January 2016 onward an increasing workload of the medical doctorsled to the decision that midwives working in our infertility centre should perform IUI procedures. The rationale behind this was to offer a more patient-centred service and all 5 participating midwives received a training course to standardise the release time with special attention to the duration of injecting. For this, a song was learned in order to define and set the pace to release the inseminate from the injection. This resulted in a slow-release sperm injection of at least 45-60 seconds. For patient comfort and compliance, the song was sung in ‘head voice’ achieving a silent and comforting atmosphere during the injection. Patient selection, cycle monitoring and the use of natural cycle or ovarian stimulation remained the same in both periods. Comparing the data and outcome results of the two study periods, the only variables that changed were the time of sperm injection (bolus versus modified slow-release) and the person who performed the IUI (doctor versus midwife). Serum ß-hCG was determined 14–16 days after IUI. Clinical pregnancy was confirmed by ultrasonography 5-6 weeks after IUI with the presence of a gestational sac and foetal heartbeat. Ethical approval for this prospective cohort study was obtained on 31 May 2011 (reference number: 13/054U).

### Statistical analysis

One of the most important assumptions of all classical statistical analyses is the assumption of independency. For the dataset considered here, however, this assumption is not fulfilled. In case of a failed first attempt, the patient probably will return for a second or third attempt. Even if the patient becomes pregnant, they will return to try and expand their family. This discloses that not all observations were independent: some observations come from the same patient, whereas other observations come from different patients. To take into account this dependency, we modelled the probability of becoming pregnant using generalised estimating equations (GEE) model (Zeger and Liang, 1986; Molenberghs and Verbeke, 2005). This model can be seen as an extension of ordinary logistic regression where the correlation between observations from the same person is taken into account. In the present study, the correlation structure was assumed to be of an ‘exchangeable’ type. GEE is known to be robust against misspecification of the working correlation structure.

Statistical significance was established at p< 0.05. As the amount of missing data was low, cycles that contained missing data were not included in the GEE analysis. All GEE analyses were done in the software package SAS ® version 9.4 for Windows (Belgium). Continuous data are presented as mean ± standard deviation (SD), whereas categorical (or categorised) data are presented in terms of the CPR ± standard error (SE).

First of all, univariate analyses were performed in order to investigate the influence of several covariates on the CPR (i.e. for each covariate separately). Covariates taken into account included female and male age (years), smoking both partners (yes or no), BMI both partners (kg/m^2^), primary/secondary infertility, cycle rank, ovarian stimulation method [natural cycle (NC), CC, hMG/rFSH], day 0 oestradiol (ng/l) and progesterone (µg/l) levels, abstinence period (days), human chorionic gonadotrophin (hCG)-insemination time interval (hours), easy or difficult insemination and sperm quality parameters [i.e. volume (ml), concentration (million/ml), total count (million), motility grade A (%), progressive motility (%), sperm morphology (% normal forms) and IMC (million)]. The main characteristics of the study population before (period 1) and after (period 2) 2016 were also compared using univariate GEE models.

All covariates that were associated with CPR in these univariate analyses (based on p < 0.20) were included in a multivariable GEE model. Backward model selection was performed, i.e. in each step the least significant (with p > 0.05) covariate was excluded from the model until only significant (p < 0.05) covariates remained. The covariate comparing both periods was not included in this model selection procedure but was added to the final model and kept if significant (p < 0.05).

## Results

Overall, 2565 IUI treatments were given to 989 sub-fertile couples. The CPR per cycle was 10.8 % (276/2551) with a twin pregnancy rate of 6.8% (19/276). Homologous IUI was performed in 594 women during 1540 cycles in period 1 and in 395 women during 1025 cycles in period 2. The pregnancy outcome was unknown in 14 patients (0.5%).

## Univariate analyses

We found that the CPR per cycle decreased significantly with advancing patient and partner age, a lower patient BMI and a smoking partner, whereas partner BMI and patient smoking did not significantly influence CPR per cycle (Table I).

**Table I:** Univariate analysis on covariates related to patient characteristics.

Parameter	CP	Total	CPR	SE	p-value
Age patient (years)					**.0025**
<30	133	942	14.1	1.1	
30-34.99	88	885	9.9	1.0	
35-39.99	41	515	8.0	1.2	
≥40	14	206	6.8	1.8	
Age partner (years)					**.0014**
<30	82	559	14.7	1.5	
30-34.99	107	890	12.0	1.1	
35-39.99	49	635	7.7	1.1	
≥40	38	465	8.2	1.3	
Smoking patient					.1058
Yes	29	338	8.6	1.5	
No	247	2213	11.1	0.7	
Smoking partner					**.0421**
Yes	60	688	8.7	1.1	
No	216	1863	11.6	0.7	
BMI patient (kg/m^3^)					**.0031**
<20	33	393	8.4	1.4	
20-24.99	124	1323	9.4	0.8	
25-29.99	76	508	15.0	1.6	
≥30	43	325	13.2	1.9	
BMI partner (kg/m^3^)					.2962
<20	6	79	7.6	3.0	
20-24.99	110	1107	9.9	0.9	
25-29.99	117	1039	11.3	1.0	
≥30	43	325	13.2	1.9	

BMI = body mass index; SE= Standard Error; CP = clinical pregnancy; CPR = clinical pregnancy rate (%).

Ovarian stimulation and SRI were the only IUI procedure-related factors significantly influencing CPR. Cycles stimulated with CC resulted in a significantly lower CPR compared with cycles stimulated with hMG or rFSH. Levels of oestradiol and progesterone on day 0 (day of ovulation triggering) and time intervals between hCG injection and insemination had no significant influence on CPR (Table II). Easy or difficult insemination, occurrence of blood loss during or after insemination, number of days of abstinence before delivery of the semen sample for IUI and rank of IUI attempt did not significantly influence CPR.

**Table II:** Univariate analysis on covariates related to the IUI procedure.

Parameter	CP	Total	CPR	SE	p-value
Stimulation					**.0032**
NC	51	433	11.8	1.6	
CC	100	1162	8.6	0.8	
HMG/rFSH	125	947	13.2	1.1	
HCG-insemination interval (h)					.0513
<15	12	148	8.1	2.2	
15-22.99	93	982	9.5	0.9	
≥23	171	1421	12.0	0.9	
Oestradiol D0 (ng/l)					.1689
12-240	81	672	12.1	1.3	
241-368	66	631	10.5	1.2	
369-533	43	524	8.2	1.2	
534-1700	61	534	11.4	1.4	
Progesterone D0 (µg/l)					.3777
<0.5	157	1340	11.7	0.9	
0.5-0.99	96	998	9.6	0.9	
1-1.49	16	157	10.2	2.4	
≥1.5	7	56	12.5	4.4	

Concentrations of oestradiol and progesterone at D0 and the HCG- insemination time interval were continuous variables, therefore P-values represent overall significance levels. Stimulation was a categorical variable and the P-value represents a significant difference in CPR between NC or CC and hMG/rFSH stimulated groups: a NC versus CC, b NC versus hMG/rFSH, c CC versus hMG/rFSH. CC = clomiphene citrate; CP = clinical pregnancy; CPR = clinical pregnancy rate (%); D0 = day 0; HCG = human chorionic gonadotropin; hMG = human menopausal gonadotropin; NC = natural cycle; rFSH = recombinant follicle-stimulating hormone, SE= Standard Error.

CPR showed a steady increase with increasing IMC values up until an IMC of 9.99 million, after which the CPR dropped. CPRs were highest when sperm progressive motility was above 32 % (p=0.03). All other sperm parameters including sperm morphology scores did not significantly influence CPR. Results from the univariate statistical analyses for covariates related to sperm quality are shown in Table III.

**Table III:** Univariate analysis of covariates related to sperm quality.

Parameter	CP	Total	CPR	SE	p-value
IMC (million)					**.0023**
<1	20	322	6.2	1.3	
1-1.99	12	165	7.3	2.0	
2-4.99	33	375	8.8	1.5	
5-9.99	70	471	14.9	1.6	
≥10	141	1218	11.6	0.9	
Concentration (million/ml)					.0633
0-4.99	4	77	5.2	2.5	
5-9.99	7	99	7.1	2.6	
10-14.99	10	124	8.1	2.5	
15-19.99	17	124	13.7	3.1	
≥20	238	2127	11.2	0.7	
Total count (million)					.1274
0.16-73.7	59	685	8.6	1.1	
73.8-143	84	643	13.1	1.3	
144-243	66	623	10.6	1.2	
244-2622	65	591	11.0	1.3	
Grade A motility (%)					.4650
0-7	63	607	10.4	1.2	
8-15	72	637	11.3	1.3	
16-24	63	654	9.6	1.2	
25-68	78	653	11.9	1.3	
Progressive motility (%)					**.0321**
<20	6	103	5.8	2.3	
20-31.99	20	271	7.4	1.6	
32-49.99	128	1029	12.4	1.0	
≥50	122	1148	10.6	0.9	
Morphology (%)					.3237
<4	125	1219	10.3	0.9	
4-5.99	62	547	11.3	1.4	
≥6	89	785	11.3	1.1	

All sperm parameters were continuous variables; therefore the P-values represents the overall significance level. CP = clinical pregnancy; CPR = clinical pregnancy rate (%); SE= Standard Error; IMC = inseminating motile count.

A summary of the univariate analyses results is shown in Table IV. Care should be taken with the interpretation of these results, because the effect of only one covariate at a time is shown and this could be influenced by other factors. Only the significant covariates (p<0.20, in bold) were taken into account for the final multivariable GEE model.

**Table IV:** IUI with partner semen: Summary of the univariate analyses. Only the significant covariates (p<0.20, in bold) are taking into account for the multivariate GEE model ((IMC =Inseminating motile count after washing procedure, D0 = day of HCG-triggering).

Parameter	p-value
Age patient	.0025
Age partner	.0014
Smoking patient	.1058
Smoking partner	.0421
BMI patient	.0031
BMI partner	.2962
Infertility (primary or secondary)	.5137
Attempt rank	.9283
Ovarian stimulation	.0032
Abstinence period (hours)	.2277
HCG-insemination interval	.0513
Oestradiol D0 (ng/l)	.1689
Progesterone D0 (µg/l)	.3777
Bloodloss ++	.3731
IMC (million)	.0023
Concentration (million/ml)	.0663
Total count (million)	.1274
Grade A motility (%)	.4650
Progressive motility (%)	.0321
Total Motile Sperm Count	.3293
Anti sperm antibodies > 50 %	.5493
Morphology (%)	.3237
SRI	.0003

## Multivariable GEE analysis

The results of the multivariable analysis (GEE model) indicate that only partner age, partner smoking, patient BMI, ovarian stimulation, IMC, and SRI significantly influenced CPR (Table V). When SRI was performed (period 2), the CPR was significantly higher compared to bolus IUI (period 1).

**Table V:** Results from the multivariate generalised estimating equation analysis.

Covariate	Parameter estimation (SE)	p-value
Midwife SRI	0.4355 (0.1352)	**.0013**
Age partner		<.0001
< 30 vs 30 - 34.99	0.3125 (0.1674)	0619
**< 30 vs 35 - 39.99**	0.8168 (0.2001)	**<.0001**
**< 30 vs ≥ 40**	0.7654 (0.2253)	**.0007**
30 – 34.9 vs 35 - <40	0.5043 (0.1882)	.0074
**30 - 34.99 vs ≥ 40**	0.4530 (0.2104)	**.0313**
35 - 39.99 vs ≥ 40	-0.0514 (0.2396)	.8303
Smoking partner	-0.3251 (0.1620)	.0447
BMI patient		**.0017**
**< 20 vs 25 - 29.99**	-0.7294 (0.2318)	**.0016**
< 20 vs 20 - 24.99	-0.1793 (0.2154)	.4051
**< 20 vs ≥ 30**	-0.6168 (0.2671)	**.0206**
**20 - 24.99 vs 25 - 29.99**	-0.5502 (0.1608)	**.0006**
**20 - 24.99 v s ≥ 30**	-0.4393 (0.2083)	**.0350**
25 - 29.99 vs ≥ 30	0.1108 (0.2490)	.6190
Ovarian stimulation		.0020
**NC vs CC**	0.4851 (0.1448)	**.0008**
NC vs hMG/rec FSH	0.0387 (0.1806)	.8305
**CC vs hMG/rec FSH**	-0.4464 (0.1826)	**.0145**
IMC		**<.0001**
**< 5 vs 5 - 9.99**	-0.8416 (0.1843)	**<.0001**
**< 5 vs ≥ 10**	-0.5163 (0.1586)	**.0011**
**5 - 9.99 vs ≥ 10**	0.3253 (0.1541)	**.0347**

(IMC = Inseminating Motile Count, BMI = Body Mass Index, NC= natural cycle, CC =clomiphene citrate, hMG = human gonadotrophines, rec FSH = recombinant FSH, SRI = slow-release intrauterine insemination, SE = Standard Error).

A significantly lower pregnancy rate was found for partners above 35 compared to partners below 35 years old, for women with a smoking partner and with a patient BMI of less than 25. Using no ovarian stimulation (natural cycle) resulted in significantly higher CPR compared with CC, while CC resulted in significantly lower CPR compared to hMG/rec FSH.

A significantly higher CPR was observed for an IMC between 5 and 10 million when compared to the <5 million and > 10 million groups. An IMC above 10 million was significantly better than an IMC below 5 million.

## Outcome results period 1 (bolus IUI) versus period 2 (modified SRI)

**Figure 1:**
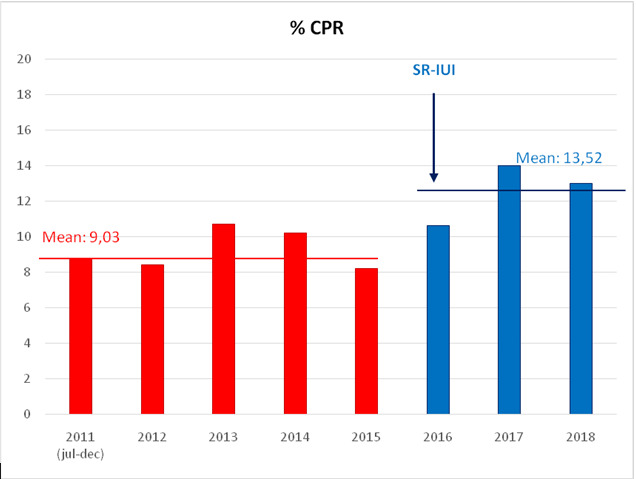
Clinical pregnancy rate per year in the period July 2011 –December 2018. From 2016 onwards SR-IUI (slow-release patient centred intrauterine insemination) was adapted.

Figure 1 shows the CPR per year in the period July 2011 –December 2018. After 2015 (period 2) a significant increase in CPR can be observed. The CPR per cycle was 9.03 % (138/1529) in period 1 compared to 13.52% (138/1022) in period 2 resulting in a significant increase of 4.5 % per cycle (p = 0.001). Per pregnancy, multiple pregnancy rate in this cohort was 6.5% (9/138) in period 1 and 7.2% (10/138) in period 2 (p = 0.81).

Considering the indication for IUI (unexplained or male subfertility) there was no difference between both study groups. A male factor was found in 48.3 % of patients (494/1022) in period 2 compared to 48.4 % (741/1529) in period 1 (p = 0.96).

The main characteristics of the study population in both periods are shown in Table VI.

**Table VI:** Main characteristics of the study population before and after 2016. Data are presented as GEE predicted mean +/- SE.

Parameter	Before 2016	After 2015	p-value
Patient characteristics			
**Age patient** (years)	31.5 +/- 0.2	33.0 +/- 0.2	**<.0001**
**Age partner** (years)	34.6 +/- 0.2	35.5 +/- 0.3	**.0070**
**BMI patient** (kg/m^3^)	24.2 +/- 0.2	24.8 +/- 0.2	**.0101**
BMI patient (kg^3^)	26.0 +/- 0.1	26.1 +/- 0.2	**.9508**
IUI procedure characteristics			
**HCG-insemination interval** (h)	21.2 +/- 0.2	25.5 +/- 0.1	**<.0001**
**Oestradiol D0** (ng/l)	415.6 +/- 8.1	354.9 +/- 8.6	**<.0001**
**Progesterone D0 (µg/l)**	0.7 +/- 0.02	0.5 +/- 0.04	**<.0001**
Sperm characteristics			
**IMC** (million)	16.8 +/- 0.7	14.8 +/- 0.7	**.0252**
Concentration (million/ml)	60.7 +/- 1.6	57.9 +/- 1.8	.1982
Total count (million)	180.9 +/- 6.2	166.7 +/- 6.1	.0926
Grade A motility (%)	17.4 +/- 0.4	17.1 +/- 0.5	.5915
**Progressive motility** (%)	47.9 +/- 0.5	45.2 +/- 0.6	**.0002**
**Morphology** (%)	4.9 +/- 0.1	4.2 +/- 0.1	**<.0001**

## Discussion

As a result of three excellent randomised prospective studies (Bensdorp et al., 2015; Tjon- Kon-Fat et al., 2015; Farquhar et al., 2018) IUI can be recommended as a valuable first-line strategy for mild male factor or unexplained infertility (Cohlen et al., 2018). Nevertheless, strategies to improve pregnancy rates per IUI treatment are urgently needed to compete with IVF/ICSI due to the increase in pregnancy rates and the decrease in ART related complications during the last decade. Most studies on IUI strategies to increase pregnancy rates deal with ovarian stimulation protocols, timing of IUI, sperm quality factors, sperm processing techniques, etc., with discouraging results.

A better selection of couples who are the best candidates for IUI can be another option. For example, in patients with mid-distal or distal unilateral tubal occlusion, a significant decrease in success rate is reported. These patients should be referred for laparoscopic assessment and in a substantial number of cases IVF instead of IUI should be the first-choice treatment (Lin et al., 2013; Berker et al., 2014). Recent reports also describe a significantly negative effect of human papilloma virus (HPV) positivity in men and/or women on clinical pregnancy rates following IUI (Depuydt et al., 2016; 2019). Therefore, HPV positive women and men should not receive IUI as a first-line treatment and a waiting period of 6 months can be recommended as HPV is a transient infection clearing spontaneously within 6-12 months in most cases (Garolla et al., 2013).

We can also try to increase success rates by changing the technique and methodology of IUI.

The slow-release insemination (SRI) instead of the regular bolus IUI injection seems to be a promising strategy to improve IUI-success. The rationale underlying SRI is that the inseminated motile spermatozoa are released into the uterus during an extended period of time with less spermatozoa expelled through the fallopian tube into the peritoneum.

In a randomised cross-over study with a Grasby type MS16 pump for 3 hours the CPR per cycle and cumulative pregnancy rate after 4 cycles improved from 6.1% to 22% and from 15.0% to 63.1%, respectively (Muharib et al., 1992). The authors hypothesised that the period of potential fertilisation might increase by injecting a persistent low concentration of spermatozoa.

Marschalek et al. (2017) published data from two pilot randomized, controlled cross-over studies, indicating a statistically significant advantage of SRI over conventional bolus IUI. To perform the slow-release injection they used a disposable EVIE syringe pump (Fertiligent, Ra’anana, Israel), a 3 ml sterile syringe (Becton Dickinson; Franklin Lakes, NJ) and a customised HSG catheter with inflatable anchor balloon at the tip (Catheter Research Inc; Indianapolis, IN).

Moreover, results of a multicentre, randomised controlled trial, comparing bolus IUI with SRI with a duration of 4 hours, were recently reported by the same authors, using the same EVIE device (Marschalek et al., 2020). At total of 182 women were randomised to receive bolus- IUI (n = 96) or SRI (n = 86). Patients who did not conceive after the first cycle switched to the alternative technique for the second cycle. Pregnancy rates following SRI and IUI showed a non-significant difference of 13.2% and 10.0% (p = 0.202). In a subgroup of women aged under 35 years, the pregnancy rate with SRI was 17% compared to 7% with bolus IUI, a significant difference (relative risk 2.33; p = 0.032). These results support the hypothesis that pregnancy rates might be improved with SRI compared to bolus IUI, especially in women aged under 35 years.

In our study and by using generalised estimating equations (GEEs) to account for clustered observations, we found a significant increase (4.5 %) in clinical pregnancy rate in period 2 (after 2015), although only one intervention parameter had been changed during that period, namely a modified slow-release injection of processed sperm instead of a bolus injection.

Comparing the patients’ characteristics in period 1 versus period 2, a significant difference in various parameters can be observed (Table VI). For all significant differences in period 2 compared to period 1, one should expect a lower CPR in period 2 (higher male and female age, lower IMC, lower progressive motility, and lower sperm morphology score). Therefore, we do not consider these differences to be of value when interpreting the differences in CPRs between both periods. The hCG-IUI time interval was significantly longer in period 2 compared to period 1. This was caused by a re-organisation in the IUI programme and inseminations were performed a bit later in the afternoon. Accordingly, results of a Cochrane review on different time intervals between hCG and IUI found that this difference does not influence pregnancy rates. They concluded that IUI should be performed between 12 to 36 hours after hCG injection, with comparable results (Cantineau et al., 2014). Therefore, we assume that this difference in timing between both groups is not important, although, it should be taken into account when planning a prospective randomised controlled trial.

Considering the limitations of this study, a distinction between the effect of a patient-centred approach, SRI and/or bolus injection can only be accomplished in a prospective randomised controlled trial. One should investigate whether increasing patient-centredness may have played a role to explain the better outcome results. Previous reports have highlighted the importance of sufficient emotional support for couples undergoing assisted reproduction (van Empel et al., 2011; Boivin et al., 2015). Most patients prefer continuity of care and do not want to be treated by too many different fertility clinic staff members. In our study midwifes were involved in the pre- treatment counselling and the follow-up of the patients during treatment (ovarian stimulation and follicle monitoring). This was not the case for most medical doctors. They occasionally had contact with the patients before the IUI was performed.

Another possible weakness of this study might be the use of CPR per cycle as the main outcome parameter instead of LBR (live birth rate) per cycle or cumulative pregnancy rate. Clarke et al. (2010) analysed 42 reviews including 654 RCTs, of which 143 (22%) reported on pregnancy and live birth rates. They concluded that the effectiveness of a treatment based on either clinical pregnancy or live birth as endpoints were de facto comparable.

This study also provides some important strengths. Firstly, the collection of data was prospective, as different patient and treatment- specific factors were recorded by means of a questionnaire (CRF) at the time of insemination (Thijssen et al., 2017). The results of the CRFs were examined by a third person on a monthly basis for possible lack of data. Secondly, the multivariable GEE analysis used in this study has a major advantage over previously used ordinary logistic regression models, as it takes into account the correlation between observations from the same patient when patients are coming back for treatment after previous failed attempts. Lastly, in period 2 compared to period 1only one study aspect changed, specifically, modified slow-release IUI with a patient-centred approach instead of regular bolus injection.

From an economical point of view, the increased use of IUI and IVF-related procedures over the past decades and the costs associated with the reimbursement of these treatments are pressing concerns to health service providers. In order to remain affordable and sustainable, we need to search for a responsible use of public funds. Our study shows that, by increasing the duration of insemination combined with a patient-centred approach, significantly better pregnancy rates can be achieved without increasing costs.

## Conclusion

The results of our prospective cohort study investigating the influence of SRI versus bolus-IUI demonstrate a significant increase in CPR in a large series of homologous inseminations.

Future prospective randomized studies should be performed to investigate whether SRI or patient-friendly measures or their combination are responsible for this improvement.
